# Lower visual field preference for the visuomotor control of limb movements in the human dorsomedial parietal cortex

**DOI:** 10.1007/s00429-021-02254-3

**Published:** 2021-03-18

**Authors:** Teresa Maltempo, Sabrina Pitzalis, Martina Bellagamba, Sara Di Marco, Patrizia Fattori, Gaspare Galati, Claudio Galletti, Valentina Sulpizio

**Affiliations:** 1 Department of Movement, Human and Health Sciences, University of Rome “Foro Italico”, Rome, Italy; 2 Department of Cognitive and Motor Rehabilitation and Neuroimaging, Santa Lucia Foundation (IRCCS Fondazione Santa Lucia), Rome, Italy; 3 Department of Biomedical and Neuromotor Sciences, University of Bologna, Bologna, Italy; 4 Department of Psychology, “Sapienza” University of Rome, Via dei Marsi 78, 00185 Rome, Italy

**Keywords:** Visuomotor control, Superior parietal lobule, Pointing, Functional magnetic resonance, HPEc

## Abstract

Visual cues coming from the lower visual field (VF) play an important role in the visual guidance of upper and lower limb movements. A recently described region situated in the dorsomedial parietal cortex, area hPEc (Pitzalis et al. in NeuroImage 202:116092, 2019), might have a role in integrating visually derived information with somatomotor signals to guide limb interaction with the environment. In macaque, it has been demonstrated that PEc receives visual information mostly from the lower visual field but, to date, there has been no systematic investigation of VF preference in the newly defined human homologue of macaque area PEc (hPEc). Here we examined the VF preferences of hPEc while participants performed a visuomotor task implying spatially directed delayed eye-, hand- and foot-movements towards different spatial locations within the VF. By analyzing data as a function of the different target locations towards which upcoming movements were planned (and then executed), we observed the presence of asymmetry in the vertical dimension of VF in area hPEc, being this area more strongly activated by limb movements directed towards visual targets located in the lower compared to the upper VF. This result confirms the view, first advanced in macaque monkey, that PEc is involved in processing visual information to guide body interaction with the external environment, including locomotion. We also observed a contralateral dominance for the lower VF preference in the foot selective somatomotor cortex anterior to hPEc. This result might reflect the role of this cortex (which includes areas PE and S-I) in providing highly topographically organized signals, likely useful to achieve an appropriate foot posture during locomotion.

**Supplementary Information:**

The online version contains supplementary material available at 10.1007/s00429-021-02254-3.

## Introduction

Humans are more efficient when performing actions towards objects presented in the lower visual field (VF) compared to the upper VF (Danckert and Goodale 2001). This view is consistent with the proposed specialization of the lower VF in the perceptual processes required for visuomotor coordination in the peripersonal space (Previc 1990; Danckert and Goodale 2003). In particular, it has been suggested that visual cues coming from the lower VF (including the vision of the moving hand and feet) might play an important role in the visual guidance of upper and lower limb movements (Graci et al. 2011) and that the higher performance observed in actions performed in the lower VF may be explained by better use of visual feedback during movement execution (Khan and Lawrence 2005).

Functional data, reviewed in Gamberini et al. (2020), suggested that the caudal aspect of the Superior Parietal Lobule (SPL), a region called area PEc, might have a role in integrating visually derived information with somatomotor signals to control and coordinate movements of both upper and lower limbs during the whole-body interaction with the environment.

In macaque, area PEc responds to stimulation of the joints of both arm and leg (Breveglieri et al. 2006, 2008) and contains visual cells sensitive to complex stimuli continuously changing in size and speed (Gamberini et al. 2018), as well as to optic flow stimuli (Raffi et al. 2002, 2008). Area PEc is specialized in the integration of hand- and eye-related information (Ferraina et al. 2001) and it is well equipped to control arm-reaching actions (Hadjidimitrakiset al. 2015; Piserchia et al. 2017). Additionally, PEc receives visual information mostly from the lower visual field, somatosensory signals from all the four limbs, and is strongly connected with cortical regions that represent the lower limbs (Nelson 1980; Kaas 1983; Bakola et al. 2010; Gamberini et al. 2018, 2020).

In human, area PEc (hPEc; Pitzalis et al. 2019) responds to both arm and leg movements (although leg movements elicit stronger activations), shows a sensitivity to self-motion compatible visual stimulation (optic flow) (Pitzalis et al. 2020) and is involved in implementing the sensorimotor transformations needed to actually grasp a visually presented object (Sulpizio et al. 2020). In good agreement with the view that hPEc is involved in limb interaction with the environment, Abdollahi et al. (2013) found that a region in the human dorsal SPL that likely includes the homologue of the monkey’s area PEc is activated when observing locomotion and, even more, when observing climbing. Finally, it was reported that a patient with a damage of the posterior part of the SPL, that likely included hPEc, showed severe impairment of body interaction with the surrounding objects (Kase et al. 1977).

Taken together, both macaque and human data seem to support the hypothesis that area PEc might support the visuomotor guidance of upper and lower limb movements, for example during complex whole-body movements such as locomotion. In this scenario, visual cues coming from the lower VF should be particularly useful for limb positioning, for example during adaptive gait or when walking on uneven terrains. However, while in macaque this hypothesis is supported by the main representation of the lower visual field in PEc (Breveglieri et al. 2008; Bakola et al. 2010; Raffi et al. 2014; Hadjidimitrakis et al. 2015; Gamberini et al. 2018), in human, a clear preference for the lower visual field in the newly defined homologue of macaque PEc (Pitzalis et al. 2019) is still lacking.

In the current study, we examined the VF preferences of area hPEc by analyzing brain activations from a visuomotor task (previously published in Pitzalis et al. 2019) implying spatially directed delayed eye, hand, and foot movements. Specifically, we tested whether the portion of the VF to which the upcoming eye/hand/foot movements were directed could differentially impact on the hPEc activity. To this aim, participants performed a short-range pointing movement towards different spatial locations within the VF by using the instructed effector. Data were thus analyzed as a function of effector and portion of VF (along both the horizontal -upper and lower- and the vertical -left and right- dimensions) to which the movement was directed.

We also examined the role of two neighboring anterior regions, defined in Pitzalis et al. (2019) as hPE (human PE, in the postcentral sulcus) and the dorsomedial portion of S-I (Somatosensory-I). These two somatic regions respond to long-range leg movements (Pitzalis et al. 2019), with hPE being also sensitive to egomotion-compatible optic flow, although at lower extent than hPEc (Pitzalis et al. 2019; Di Marco et al. 2021). We recently found further positive evidence of the presence of visual responses in these somatic regions. Specifically, we described hPE and S-I, as involved, together with area hPEc, in controlling changing direction of self-motion during locomotion, being activated by active leg movements and by locomotion-compatible path curvature from optic flow (Di Marco et al. 2021).

In this study, we observed that hPEc was significantly more activated by limb movements directed towards the lower relative to the upper VF, thus suggesting that this area might play an important role in processing visual information to guide body interaction with the external environment, including locomotion. Additionally, we found that the most anterior portion of the SPL, i.e., the cortical territory hosting foot-selective representations corresponding to the somatic areas hPE and S-I, showed a lower VF preference only for foot and only for movements directed in the contralateral visual hemifield, thus suggesting its possible role in the visuomotor control of foot movements.

## Methods

### Participants

The present study is based on a reanalysis of BOLD data from a sub-sample of subjects (*N* = 18, 10 females, mean age 25.22 years, SD 3.39 years) who participated to a previous study from our lab (Pitzalis et al. 2019). Two subjects of the original sample were excluded because, based on the post-scanning debriefing, they reported having difficulty in rotating their foot down. All participants had normal or corrected-to-normal vision and no previous history of psychiatric or neurologic disease. Hand and foot right-dominance were tested by the Edinburgh handedness inventory (Oldfield 1971). All volunteers had given their written informed consent to participate, and the original studies had been approved by the research ethics committees at Fondazione Santa Lucia in Rome, according to the Declaration of Helsinki.

### Stimuli and experimental paradigm

**Fig. 1:**
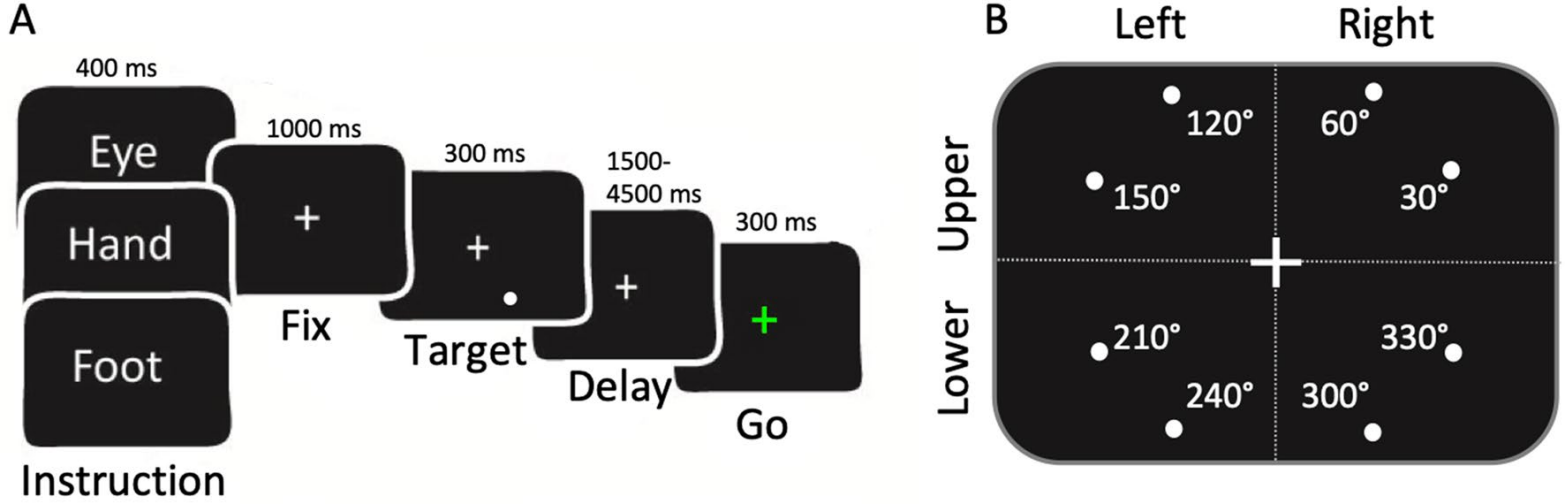
Visuomotor task*.* **a** Description of the pointing task: subjects alternated blocks of memory delayed saccadic eye/hand/foot pointing movements to peripheral visual targets with passive fixation blocks (not shown). **b** Schematic view of how the eight possible target locations are arranged with respect to the vertical (lower, upper) and horizontal (left, right) dimensions of VF. Angular location of each target (not shown to the participant) is also shown. Further details about target positions are available in Supplementary Table 1

Each participant underwent (1) a localizer session, consisting in active movements of the inferior (leg) and superior (arm) limbs (somatomotor task), designed to maximally activate leg and arm movement-related cells in the dorsomedial SPL and (2) a visuomotor task (delayed eye/hand/foot pointing; Fig. 1) designed to reveal the responsiveness to spatially directed pointing movements and to isolate effector-selective representations in the parietal cortex.

1. Somatomotor task. This task was described in the original paper by Pitzalis et al. (2019). Briefly, subjects were instructed to execute long-range arm and leg movements, designed to maximally stimulate limbs joints and activate somatomotor neurons. Each block started with a written instruction (“FIX”, “LEG” or “ARM”) presented for 400 ms at the center of the screen to inform the participant on the task to be performed. During fixation blocks, subjects were asked only to maintain fixation throughout the block. In leg and arm blocks, the white fixation cross turned red for 300 ms (warning signal for the movement preparation) and, after a variable delay (750, 1000, 1250, 1500 ms), turned green (go signal) for 4 s, instructing participants to execute a 4 s sequence of limb movement while keeping central fixation. Each scan consisted of four consecutive trials for each block with seven arm and seven leg movement blocks lasting 20.5 s each, arranged in a pseudo-random sequence. These blocks were interleaved with 14 fixation periods of variable duration (12, 14 or 16 s). Before entering the scanner, participants were trained to correctly perform the task; then, in the scanner, they underwent a short warm-up phase to familiarize themselves with the set-up for leg movements to execute the movements as fluid as possible.

2. Visuomotor task. A detailed description of this task is provided in Pitzalis et al. (2019; see also Fig. 1). Each trial began with observers maintaining central fixation while holding a button down with their right index and a foot pedal down with their right foot. Each block started with a written instruction (“Fix”, “Eye”, “Hand”, and “Foot”) appearing for 400 ms at the center of a screen to inform about the task to be performed, followed by four trials (Fig. 1a). On each trial, a peripheral target (a white dot of diameter 0.9° in size) indicating the location for the upcoming movement appeared for 300 ms in one of eight different angular positions at an eccentricity of 4° of visual angle (Fig. 1b). For a more detailed description of the target location see Supplementary Table 1. Targets were distributed along a circle whose center corresponded to the center of the screen (i.e., the fixation point). Starting from the right horizontal meridian and following a counterclockwise direction, the target could appear at 30°, 60°, 120°, 150°, 210°, 240°, 300°, 330° of angular position, i.e., four times in the upper VF (30°, 60°, 120°, 150°) and four times in the lower VF (210°, 240°, 300°, 330°). Target which appeared at 30°, 60°, 300°, 330° fell in the right visual field, while target which appeared at 120°, 150°, 210°, 240°, fell in the left visual field. Note that the target never appeared at 0°, 90°, 180°, 270° since these locations fell along the vertical and horizontal meridians. During each block target locations appeared in a random order. After a variable delay (1.5, 2.5, 3.5, or 4.5 s), the fixation point turned green (go signal) for 300 ms and participants either released the button/pedal and rotated their wrist/ankle (without moving the shoulder/leg) to point toward the remembered target location with their right index/toe while keeping central fixation (hand/foot pointing blocks) or moved the eyes while continuing to hold the buttons (eye blocks). Subjects were instructed to immediately return to resting position after movement execution. Visual stimuli were back projected onto a screen positioned behind the subjects’ head and visible through a mirror above the head coil. In this way, targets appeared as if they were positioned in front of the subjects, just above their heads. To allow participants to rotate the limb in the direction cued by the target, we placed a cylindric cushion to support their wrist/ankle so as they could rotate their right index/big toe towards all the possible ‘remembered’ target locations. Before starting the experiment, participants performed a short warm-up within the scanner to familiarize with the task and the movement directions. Note that we asked participants to perform very short-range pointing movements and they reported being able to rotate their joints in order to point towards the spatial locations previously indicated by the target. Each scan included 4 eye, 4 hand and 4 foot-pointing blocks lasting 18 s each, arranged in a pseudo-random sequence and interleaved with 11 fixation periods of variable duration (12, 14 or 16 s).

### Apparatus and procedure

Functional images were acquired using a 3 T Siemens Allegra MR system (Siemens Medical systems, Erlangen, Germany) equipped for echo-planar imaging with a standard head coil and operating at the Neuroimaging Laboratory, Foundation Santa Lucia. Visual stimuli were presented by a control computer located outside the MR room, running in-house software (Galati et al. 2008) implemented in MATLAB (The MathWorks Inc., Natick, MA, USA). We used an LCD video projector with a customized lens to project visual stimuli to a projection screen positioned at the back of the MR tube. The timing of presentation of each stimulus was controlled and triggered by the acquisition of fMRI images.

We used blood-oxygenation level-dependent imaging (Kwong et al. 1992) to acquire echo-planar functional MR images (TR = 2 s, TE = 30 ms, flip angle = 70◦, 64 × 64 image matrix, 3 × 3 mm in-plane resolution, 30 slices, 2.5 mm slice thickness with no gap, ascending excitation order) in the AC–PC plane. Images were acquired starting from the superior convexity and extended ventrally so that to include the whole cerebral cortex, excluding only the ventral portion of the cerebellum. For each participant we also acquired a three-dimensional high-resolution anatomical image (Siemens MPRAGE sequence, TR = 2 s, TE = 4.38 ms, flip angle = 8◦, 512 × 512 image matrix, 0.5 × 0.5 mm in-plane resolution, 176 contiguous 1 mm thick sagittal slices). For each scan, we discarded the first four volumes to achieve steady-state, and the experimental task was initiated at the beginning of the fifth volume.

In separate days, each subject completed two or three 526-s-long scans of the somatomotor task and two or three 402-s-long scans of the visuomotor task and one anatomical scan. During both the somatomotor and visuomotor tasks, subjects’ movements were supported by a dedicated MRI-compatible setup allowing subjects to perform controlled leg/foot movements (see Pitzalis et al. 2019 for a detailed description). To minimize movements during the scans, subjects’ head was stabilized with foam padding and with a chin rest mounted inside the head coil.

### Image processing and analysis

Images were preprocessed and analyzed using SPM12 (Wellcome Department of Cognitive Neurology, London, UK) and FreeSurfer 5.1 (http://surfer.nmr.mgh.harvard.edu/).

We first analyzed structural images following the “recon-all” fully automated processing pipeline implemented in FreeSurfer 5.1. This procedure allows us to obtain a surface representation of each individual cortical hemisphere in a standard space after performing intensity correction, transformation to Talairach space, normalization, skull-stripping, subcortical and white-matter segmentation, surface tessellation, surface refinement, surface inflation, sulcus-based nonlinear morphing to a cross-subject spherical coordinate system, and cortical parcellation (Dale et al. 1999; Fischl et al. 1999a,b; Desikan et al. 2006). The resulting surface reconstructions were transformed to the symmetrical FS-LR space (Van Essen et al. 2012) using tools in the Connectome Workbench software (https://www.humanconnectome.org/software/get-connectome-workbench), resulting in surface meshes with approximately 74 K nodes per hemisphere.

Functional images were realigned within and across scans to correct for head movement and coregistered with structural MPRAGE scans using SPM12 (Wellcome Department of Cognitive Neurology, London, UK). Functional data were then resampled to the individual cortical surface using ribbon-constrained resampling as implemented in Connectome Workbench (Glasser et al. 2013) and finally smoothed along the surface with an iterative procedure emulating a Gaussian kernel with a 6 mm full width at half-maximum (FWHM).

Functional images were then analyzed for each participant separately on a vertex-by-vertex basis, according to the general linear model (GLM). Separate regressors were included for each combination of effector (eye, hand, foot), and target spatial position along both the vertical (upper, lower) and horizontal dimension (left, right), yielding parameter estimates for the average hemodynamic response evoked by each trial type. We modelled the whole-time interval from the target presentation to the end of the trial. We did not explicitly modeled blocks of fixation as GLM regressors that were rather treated as part of the residual variance. To reduce motion-induced noise, framewise displacement values (FD, Power et al. 2012), indicating the amount (in mm) of head movement relative to the previous time point, were also included in the model as nuisance regressors.

The main corpus of analyses was conducted on three independently defined, theoretically motivated, regions of interest (ROIs; see below). These ROIs were defined only on the left hemisphere to account for the fact that participants used their right limb effector during both the localizer scans (somatomotor task) and the main experiment (visuomotor task). For each participant and region, we computed a regional estimate of the amplitude of the hemodynamic response, obtained by entering a spatial average (weighted for the most activated nodes within the region) of the pre-processed time series into the individual GLMs. Regional hemodynamic responses were thus analyzed through a series of one sample t-tests, assessing for each condition the presence of a reliable activation. This step was essential to establish which effector(s) the region was sensitive to. We applied a Bonferroni correction for multiple comparisons (*p* = 0.05/*N* = number of conditions). When the region was significantly activated by one or more effectors, as a second step, we analyzed the BOLD signal change as a function of the experimental conditions by means of repeated-measure ANOVAs. For these analyses, we used a Bonferroni adjustment to create confidence intervals for all the pairwise differences between the factor levels.

For completeness, we also conducted some whole-brain analyses. Parameter estimated images from each participant and condition entered a group analysis where subjects were treated as a random effect. Group-level statistical parametric maps were obtained through a factorial combination of effector (foot, hand, eye), vertical dimension (lower and upper) and horizontal dimension (left and right) of VF to explore factor main effects and interactions. Further parametric maps were formed through one-sample t tests, comparing signal in eye, hand and foot conditions to the baseline (fixation). We thus conducted three conjunction null analyses (Nichols et al. 2005) to reveal any common activation between each effector-specific map (foot > fixation; hand > fixation; eye > fixation) and the lower > upper map. For all the above-described comparisons, we created several statistical parametric maps, each of them thresholded at *p* < 0.05 FDR-corrected at the cluster level, with a cluster-forming threshold of *p* < 0.001 uncorrected. Only statistical maps that survived this threshold were reported. Statistical map describing the main effect of effector was not shown since it was already described in Pitzalis et al. (2019).

### Regions of interest (ROIs)

We mainly focused our analyses on area hPEc, which we previously described as activated by limb movements and hypothesized to be relevant in the visual guidance of the body-to-environment interaction (see Pitzalis et al. 2019). We also investigated the two neighboring leg-related areas, hPE and S-I, which we previously described as potentially implicated, although at a lower extent with respect to hPEc, in the visual control of locomotion, being activated by both leg movements and self-motion compatible optic flow (Pitzalis et al. 2019; Di Marco et al. 2021). Each ROI was defined by analyzing localizer imaging scans (somatomotor task). Arm and leg blocks were modeled as box-car functions, convolved with a canonical hemodynamic response function. As described in Pitzalis et al. (2019), these three leg-related regions were defined by isolating all the activation peaks in the dorsomedial somatosensory/parietal cortex as resulting from the leg > fixation contrast map. As also detailed in Pitzalis et al. (2019), the three regions were anatomically located as follow: hPEc (identified in 15/18 hemispheres) was located in the anterior part of the dorsal precuneus, hPE (identified in 17/18 hemispheres) was located on the exposed dorsomedial surface of the anterior SPL, right over the dorsal tip of the cingulate sulcus, and S-I (identified in 18/18 hemispheres) was located on the medial portion of the brain surface, anterior to the dorsal tip of the cingulate sulcus and posterior to the dorsal tip of the central sulcus. All these ROIs were defined on the surface cortical reconstruction as automatically obtained by FreeSurfer software package.

## Results

### Lower VF preference for limb movements in hPEc

**Fig. 2:**
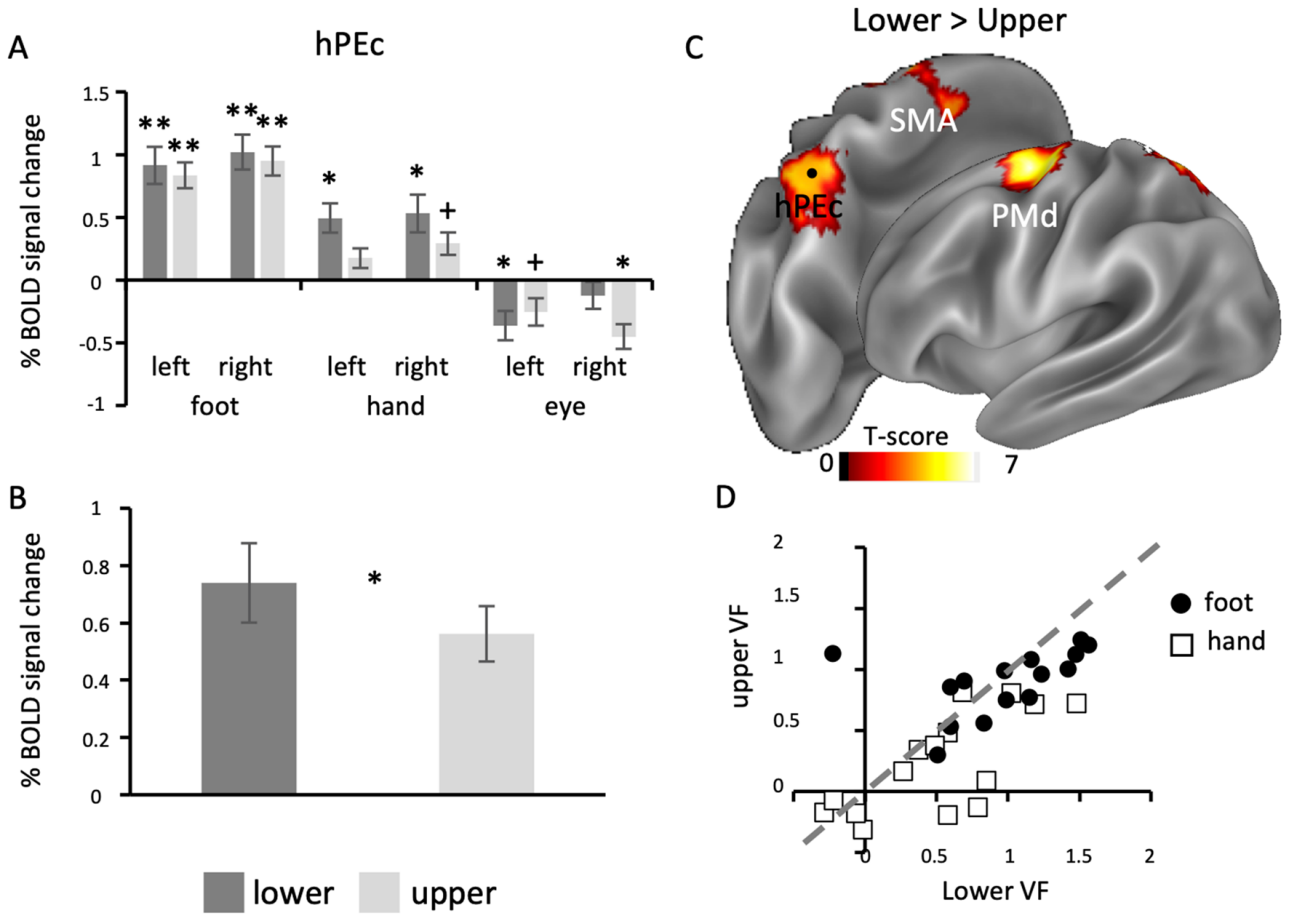
Lower VF preference for foot and hand pointing in area hPEc. **a** The plot shows the average of BOLD signal change ($\pm$ standard error) for each combination of effector (foot, hand, eye), vertical (lower, upper) and horizontal dimension (left, right) of VF in area hPEc. Asterisks refer to *t *test versus zero. *p < 0.01; **p < 0.001; +  < 0.05, Bonferroni-uncorrected. **b** The plot shows the average of BOLD signal change ($\pm$ standard error) as a function of vertical dimension (lower, upper) of VF in area PEc, collapsed across effector and horizontal dimension of VF. The plot shows the hPEc activity across the two effectors (foot and hand) showing positive (and significant) responses. Asterisk indicates the main effect of the vertical dimension (lower > upper: *p < 0.05). **c** Whole-brain activation map associated to the lower > upper contrast, displayed on the lateral and dorsomedial views of the inflated Conste69 atlas (Van Essen et al. 2012) of the left hemisphere. The center of mass of the hPEc ROI is marked by a black spot. Anatomical labels are as follows: *SMA* supplementary motor area, *PMd* dorsal premotor cortex. **d** The plot shows the estimated percent BOLD signal change for each subject in area hPEc as a function of its response to the portion of VF (lower = *X* axis; upper = *Y* axis) to which the hand- and foot-pointing movements were directed

To test the presence of positive and reliable activation within area hPEc for each combination of effector (foot, hand, eye), vertical (lower, upper) and horizontal (left, right) dimension of VF, we conducted a series of one tail *t *tests against zero. All the reported results are corrected for multiple comparisons using the Bonferroni method (see the Method section), except when differently specified. The bar graphs in Fig. 2a show the BOLD percent signal change as a function of these three factors in area hPEc. We observed significant positive responses in all conditions implying foot pointing (foot lower left: *T*_14_ = 8.30; *p* = 8.88 × 10^–7^; foot lower right: *T*_14_ = 7.22; *p* = 4.41 × 10^–8^; foot upper left: *T*_14_ = 11.25; *p* = 2.13 × 10^–8^; foot upper right: *T*_14_ = 11.19; *p* = 2.27 × 10^–8^). Other positive responses were observed in the conditions implying hand pointing, although at different extent. We observed significant positive responses when the hand pointing was directed towards the lower visual field (hand lower left: *T*_14_ = 3.690; *p* = 2.421 × 10^–3^; hand lower right: *T*_14_ = 4.168; *p* = 9.485 × 10^–4^). In the upper visual field, we observed a significant, but Bonferroni uncorrected, response when the hand pointing was directed toward the right (contralateral) hemifield (*T*_14_ = 2.853; *p* = 0.013), and a marginally significant effect when the hand pointing was directed toward the left (ipsilateral) hemifield (*T*_14_ = 2.080; *p* = 0.056). For the eye condition, only negative responses were observed (− 4.933 $\le$ *T*_14_ $\ge$ − 1.573; 2.202 × 10^–4^ $\le$ Ps $\ge$ 0.138), as previously reported in Pitzalis et al. (2019).

Further, to reveal any significant difference among conditions, we analyzed the hPEc activity through an effector (foot, hand) by vertical dimension (lower, upper) by horizontal dimension (left, right) repeated-measure ANOVA. We did not include the eye conditions since hPEc did not show a preference for this effector (see above). The ANOVA showed a main effect of effector (*F*_1,14_ = 16.892; *p* = 0.001; *η*_*p*_^2^ = 0.547) indicating that hPEc showed a stronger response to foot compared to hand pointing, as previously reported in Pitzalis et al. (2019). In addition, we observed a main effect of the vertical dimension of VF (*F*_1,14_ = 5.203; *p* = 0.039; *η*_*p*_^2^ = 0.271), indicating that hPEc exhibited a stronger response for actions directed towards targets located in the lower compared to the upper VF (see Fig. 2B). We found no significant effect of the horizontal dimension of VF (*F*_1,14_ = 3.775; *p* = 0.072; *η*_*p*_^2^ = 0.212) neither a significant interaction among factors (effector by vertical dimension: *F*_1,14_ = 2.142; *p* = 0.165; *η*_*p*_^2^ = 0.133; effector by horizontal dimension: *F*_1,14_ = 0.202; *p* = 0.660; *η*_*p*_^2^ = 0.014; vertical by horizontal dimension: *F*_1,14_ = 0.184; *p* = 0.675; *η*_*p*_^2^ = 0.013; effector by vertical by horizontal dimension: *F*_1,14_ = 0.138; *p* = 0.716; *η*_*p*_^2^ = 0.010). Note that the absence of significant interactions between effector and dimensions of VF ensures that the observed preference for the lower VF of the vertical dimension did not depend on the specific effector used, i.e., on the specific movement biomechanics, but rather on visuospatial features as the spatial location towards which the upcoming pointing movement was directed. Also the whole-brain analysis confirms this view (see below), as area PEc was not activated neither by the effector by vertical dimension interaction nor by the effector by vertical by horizontal dimension interaction.

To further explore the lower VF preference in area hPEc, we complemented the group analysis with individual data. Figure 2d shows the estimated percent BOLD signal change for each subject in area hPEc as a function of its response to the portion of VF (lower = X axis; upper = Y axis) to which the hand- and foot-pointing movements were directed. We observed that most of the subjects exhibited higher activation for targets located in the lower VF (as compared to those located in the upper VF), as indicated by the distribution of data. Indeed, data points are mainly located below the main diagonal, implying a lower VF preference for both hand and foot-pointing. In particular, 80% (12/15) of subjects showed a lower > upper trend during hand pointing and 73% (11/15) of them during foot pointing.

**Fig. 3:**
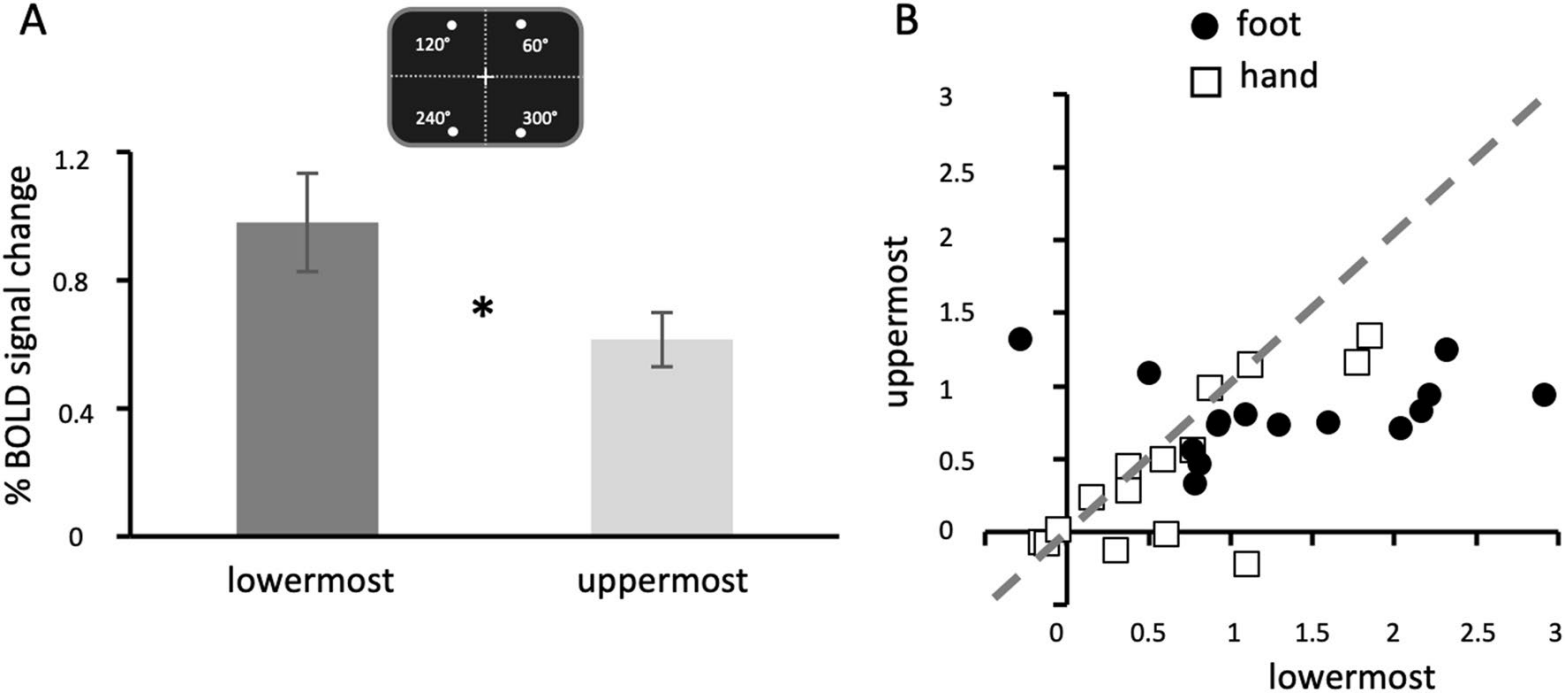
Elevation effect in area hPEc during both hand and foot pointing. **a** The plot shows the average of BOLD signal change ($\pm$ standard error) as a function of target elevation within the VF (lowermost, uppermost) in area hPEc, collapsed across effectors (hand and foot). For illustrative purpose, a schematic view of the most/less elevated target locations is displayed. Angular locations (not shown to the participant) are also displayed. **b** The plot shows the estimated percent BOLD signal change for each subject in area hPEc as a function of its response to the less elevated targets of the lower VF (*X* axis) and to the most elevated targets of the upper VF (*Y* axis) during both hand- and foot-pointing movements

Further regional analyses were conducted to account for the possibility that the lower VF preference is even stronger when comparing the hPEc activity elicited by the less elevated targets (within the lower VF) with that elicited by the most elevated ones (within the upper VF). To this aim, we restricted our analysis to the following target locations: 60°, 120°, 240° and 300° of angular position (see Figs. 1b, 3a). Targets located at 240° and 300° corresponded to the less elevated target positions while targets located at 60° and 120° corresponded to the most elevated target positions. We re-analyzed the hPEc activity through a 2 × 2 ANOVA, with effector (foot, hand) and target elevation (lowermost, uppermost) as factors. As above, we found a main effect of effector (*F*_1,14_ = 12.833; *p* = 0.003; *η*_*p*_^2^ = 0.478; foot pointing > hand pointing) as well as a main effect of target elevation (*F*_1,14_ = 7.282; *p* = 0.017; *η*_*p*_^2^ = 0.342), indicating higher activation for lowermost targets as compared to the uppermost ones (see Fig. 3a). We found no significant interaction between the two factors (*F*_1,14_ = 1.787; *p* = 0.203; *η*_*p*_^2^ = 0.113). Figure 3b shows the estimated percent BOLD signal change for each subject in area hPEc as a function of its response to the lowermost targets (X axis) and to the uppermost ones (Y axis) within the VF during both hand- and foot-pointing movements. The percentage of subjects showing a preference for movements directed towards the less elevated target locations is 53% (8/15) for hand pointing and 87% (13/15) for foot pointing. This seems to suggest that the elevation-dependent effect in area hPEc is mainly triggered by the foot effector (although the ANOVA did not reveal any effector by VF interaction), in line with our conclusion that this area has a role in the visuomotor control, especially during foot-related action like locomotion.

In summary, beyond observing the presence of both hand- and foot-related representations (with a preference for the latter), as already showed by Pitzalis et al. (2019), we found that area hPEc was preferentially activated by hand and foot pointing directed to visual target located in the lower compared to the upper VF.

Beyond the regional approach, we also conducted a whole-brain analysis to have a general picture of all the brain regions activated by the lower > upper contrast. Figure 2c shows the group activation map overlaid onto atlas Conte69 and rendered in two different inflated views (dorsomedial and lateral). In agreement with the regional analysis, we found a prominent focus of activation within the dorsomedial SPL, in a region of the precuneate cortex that well corresponds to the area hPEc (see black spot indicating the center of mass). This lower > upper map also includes a frontal activation, extending anteriorly to the precentral gyrus within the dorsal premotor area (PMd), in a portion of cortex well corresponding to the pointing-selective frontal reach region described by Tosoni et al. (2008). This frontal activation extends medially so as to include the supplementary motor area (SMA).

In line with the regional analysis, we found that the lower > upper preference observed in area hPEc was effector-independent. Indeed, whole-brain activation maps showing the effector by vertical dimension interaction (see Supplementary Fig. 1A) and the effector by vertical by horizontal dimension interaction (see Supplementary Fig. 1B) did not reveal any activation in the cortical territory hosting area hPEc. Supplementary Fig. 1A shows indeed that fMRI activation was mainly observed in the bilateral supramarginal gyrus, in the ventromedial visual areas and around the posterior segment of IPs (extending into the middle occipital gyrus) of the right hemisphere. Similarly, the significant three-way interaction map (see Supplementary Fig. 1B) did not include the precuneus and hPEc, but rather an extended fronto-parietal network mainly involving the lateral cortical surface. Although these two interaction maps are not informative about the effects’ direction in each activated cluster, they are effective in showing that the lower > upper preference observed in area hPEc is effector-independent due to the absence of a recruitment of area hPEc in both maps. This result has important implication for the result interpretation in that it greatly reduces the impact of alternative hypothesis based on the motor components of the performed action (see discussion).

Overall, both regional and whole-brain analyses indicated that area hPEc shows a clear preference for limb movements directed towards the lower VF and that this preference is independent of the specific effector used.

### Conjunction between lower VF preference and effector preference

**Fig. 4:**
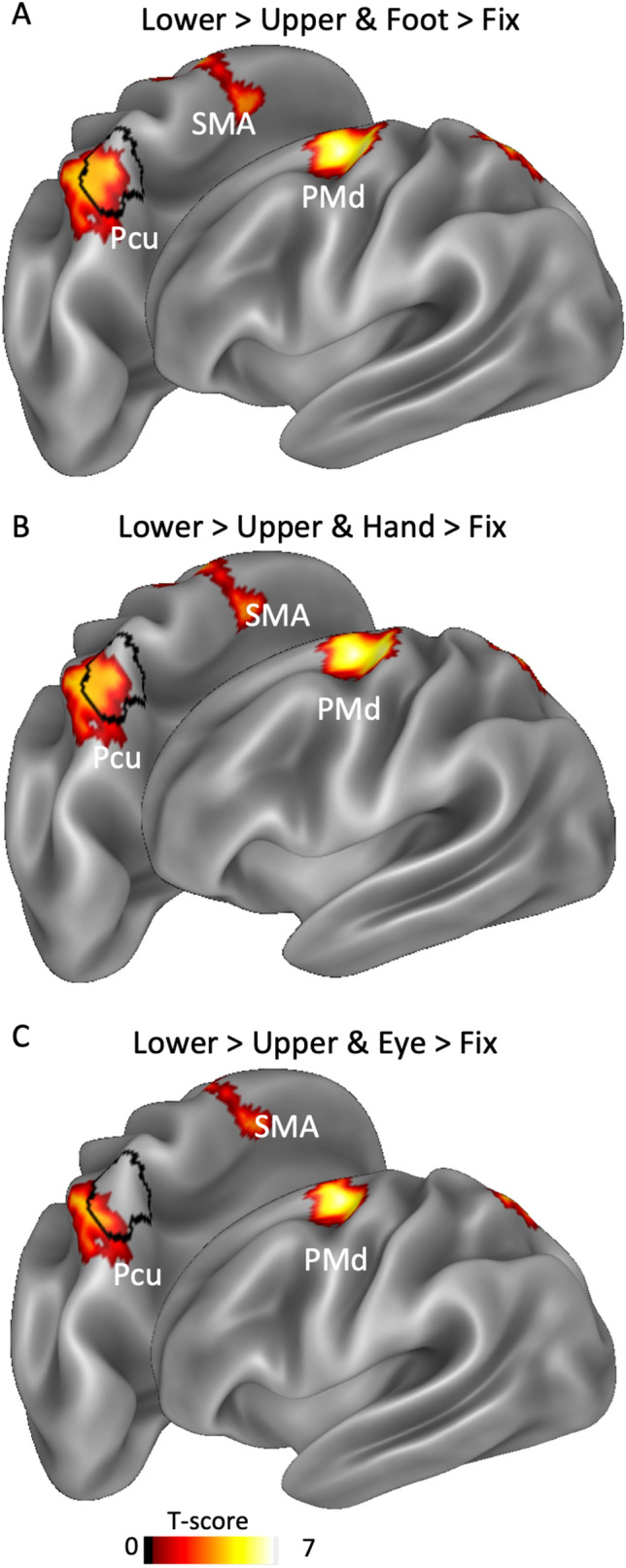
Conjunction maps. Group-based activation as resulting from the conjunction analyses between the lower > upper map and each effector-specific map, i.e., foot > fixation (**a**), hand > fixation (**b**) and eye > fixation (**c**). The borders of the hPEc ROI is marked in black. *Pcu* precuneus. Other labels as in Fig. 2

To further explore the lower VF preference in relation to the effector preference, we also conducted three conjunction analyses. Figure 4 shows the cortical regions commonly activated by the lower > upper map and each effector-specific map (A: foot > fixation; B: hand > fixation; C: eye > fixation). This analysis revealed very similar results, except for the lower > upper and eye > fixation map. In particular, although the lower > upper map was completely included in both foot- (Fig. 4a) and hand-related (Fig. 4) maps, thus confirming the hPEc involvement in representing both limbs and the lower VF, this was not the case of the lower > upper and eye > fixation map (Fig. 4c), where the hPEc territory was for the most part unresponsive. A similar trend was observed also in the frontal activation (PMd), where the lower > upper map extends more dorsally and posteriorly (up to the anterior bank of the central sulcus) in both foot- (Fig. 4a) and hand-related (Fig. 4b) maps than in eye-related map (Fig. 3c).

In summary, this analysis confirms that area hPEc contains both foot and hand (but not eye) representations overlapped with a lower VF preference for those actions.

### Lower VF preference for foot pointing in hPE and S-I

Beyond hPEc, we also investigated the two neighboring leg-related areas, hPE and S-I, which we have already described as potentially implicated, although at a lower extent with respect to hPEc, in the visual control of locomotion, being activated by both leg movements and self-motion compatible optic flow (Di Marco et al. 2021).

**Fig. 5:**
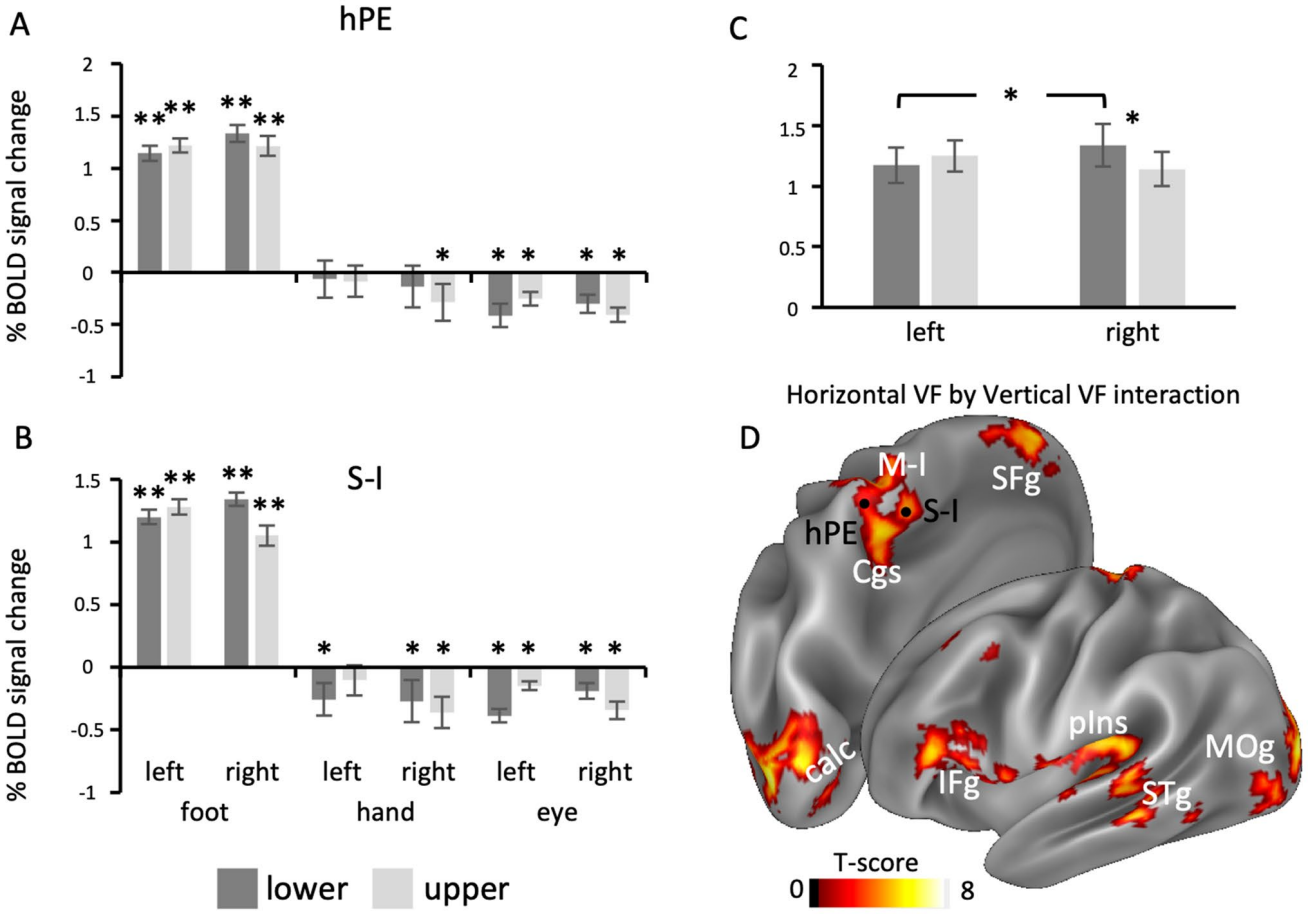
Lower VF preference for foot pointing in hPE and S-I. **a**, **b** The plots show the average of BOLD signal change( $\pm$ standard error) for each combination of effector (foot, hand, eye), vertical (lower, upper) and horizontal dimension (left, right) of VF in areas hPE (**a**) and S-I (**b**). Asterisks refer to *t *test versus zero. *p < 0.01; **p < 0.001; +  < 0.05, Bonferroni-uncorrected. **c** The plot shows the average of BOLD signal change ($\pm$ standard error) as a function of vertical (lower, upper) and horizontal dimension (left, right) of VF across the two ROIs. The plot shows the activity in the foot effector, which is the only one showing a positive (and significant) response. Asterisk indicates the interaction between vertical and horizontal dimensions of VF (lower > upper in the right VF: *p < 0.05; right > left in the lower VF: *p < 0.05). **d** Whole-brain activation map associated to the interaction contrast (right > left in the lower VF and left > right in the upper VF also corresponding to lower > upper in the right VF and upper > lower in the left VF), displayed on the lateral and dorsomedial views of the inflated Conste69 atlas (Van Essen et al. 2012) of the left hemisphere. The centers of mass of ROIs hPE and S-I are marked by a black spot. Anatomical labels are as follows: *M-I* primary motor cortex, *SFg* superior frontal gyrus, *Cgs* cingulate gyrus, *IFg* inferior frontal gyrus; *pIns* posterior insula, *STg* superior temporal gyrus, *MOg* middle occipital gyrus, *calc* calcarine fissure

As a first step, we conducted a series of one tail *t* tests against zero to reveal the presence of positive and reliable activations within both hPE and S-I for each combination of effector (foot, hand, eye), vertical (lower, upper) and horizontal (left, right) dimensions of VF. The bar graphs in Fig. 5a, b show the BOLD percent signal change as a function of these three factors in both area hPE (5a) and area S-I (5b). In both regions, we observed the presence of positive and significant activation only during foot pointing (hPE: foot lower left: *T*_16_ = 6.802; *p* = 4.244 × 10^–6^; foot lower right: *T*_16_ = 7.045; *p* = 2.765 × 10^–6^; foot upper left: *T*_16_ = 8.574; *p* = 2.230 × 10^–7^; foot upper right: *T*_16_ = 7.269; *p* = 1.876 × 10^–6^; S-I: foot lower left: *T*_17_ = 9.977; *p* = 1.599 × 10^–8^; foot lower right: *T*_147_ = 8.622; *p* = 1.295 × 10^–7^; foot upper left: *T*_17_ = 11.455; *p* = 2.041 × 10^–9^; foot upper right: *T*_17_ = 9.088; *p* = 6.168 × 10^–8^). For both hand and eye conditions, only negative responses were observed (− 7.830 $\le$ *T*_16_ < *df* < 17 $\ge$ − 1.346; 4.882 × 10^–7^ $\le$ Ps $\ge$ 0.197), as previously reported in Pitzalis et al. (2019).

Based on the hPE and S-I selectivity for foot pointing, we tested the VF preference only within the foot effector. We thus conducted a 2 by 2 by 2 ANOVA, with ROI (hPE and S-I), vertical (lower, upper) and horizontal (left, right) dimensions as repeated measures. We found no significant effects of ROI (*F*_1,16_ = 0.000; *p* = 0.991; *η*_*p*_^2^ = 0.000), vertical dimension (*F*_1,16_ = 0.619; *p* = 0.443; *η*_*p*_^2^ = 0.037) and horizontal dimension (*F*_1,16_ = 0.491; *p* = 0.494; *η*_*p*_^2^ = 0.030), neither the ROI by vertical dimension (*F*_1,16_ = 1.616; *p* = 0.222; *η*_*p*_^2^ = 0.092) nor the three-way interaction (*F*_1,16_ = 1.844; *p* = 0.193; *η*_*p*_^2^ = 0.103). We found a significant interaction between ROI and horizontal dimension (*F*_1,16_ = 8.674; *p* = 0.010; *η*_*p*_^2^ = 0.352). However, Bonferroni-corrected post hocs did not reveal significant differences among factor levels but only a marginal significance preference for the right vs left VF (*p* = 0.072) in area hPE. Interestingly, we found a significant interaction between the two VF dimensions (*F*_1,16_ = 9.087; *p* = 0.008; *η*_*p*_^2^ = 0.362), indicating a stronger activation for actions directed towards targets located in the lower compared to the upper VF, but only in the right (contralateral) hemifield (*p* = 0.029; see Fig. 5c). Post hoc comparisons also revealed a preference for the right vs left VF within the lower VF (*p* = 0.020).

Importantly, we excluded the possibility that the observed interaction between vertical and horizontal dimensions of VF depended on differences in movement biomechanics rather than on the spatial location towards the upcoming pointing movement was directed. Indeed, even if the regional analysis was focused on the foot effector, the whole-brain analyses, which were conducted on all the three effectors, ruled out this possibility, being areas hPE and S-I activated by the interaction between vertical and horizontal dimensions (see below), but not by the effector by vertical dimension interaction neither by the effector by vertical by horizontal dimension interaction (see Supplementary Fig. 1). This suggests that the observed effect was independent of the effector used and thus of the biomechanics of the specific movement performed.

Figure 5d shows the whole-brain activation map showing the interaction between vertical and horizontal dimensions of VF (*right* > *left in the lower VF and left* > *right in the upper VF* or *lower* > *upper in the right VF and upper* > *lower in the left VF*). In agreement with the regional analysis, this whole-brain analysis revealed the involvement of the medial somatomotor cortex which includes S-I and partially the neighboring hPE. Beyond these regions, we also observed a significant interaction between the two dimensions of VF in the adjoint primary motor cortex (M-I) and, more ventrally, in the posterior dorsal tip of the cingulate sulcus. Significant effects were also observed in other motor-related regions, such as the posterior insula and the inferior frontal gyrus (mainly involving the pars triangularis, BA45) and in the medial portion of the superior frontal gyrus. Other significant activations were found in more posterior regions such as the calcarine fissure and the surrounding striate and extrastriate visual areas, the adjoint middle occipital gyrus and the cortical territory around the superior and inferior temporal sulcus.

## Discussion

In the present study, we tested whether the activity of area hPEc, together with that of the anterior SPL territory hosting the two leg-related sensorimotor regions (hPE and S-I), are preferentially activated for limb actions directed towards targets presented in the lower VF than in the upper VF, as suggested by the existence of a functional advantage for the lower VF in visuomotor control (Previc 1990; Danckert and Goodale 2003). To this aim we considered a visuomotor task implying eye, hand, and foot pointing movements, that allowed to check possible differences among the used effectors. We thus analyzed data as a function of different target locations towards upcoming movements were planned and executed.

### Lower VF preference for foot and hand pointing in area hPEc

Our key finding concerns the sensorimotor area hPEc. We observed the presence of asymmetry in the vertical dimension of the VF in area hPEc, being this area, more strongly activated by limb actions directed to targets located in the lower compared to the upper VF. Interestingly, the described effect is even stronger for the less elevated targets within the lower VF as compared to the most elevated ones within the upper VF, thus strengthening the idea that area hPEc prefers limb actions triggered by targets located in the lower VF.

This result mirrors the functional profile observed in the corresponding macaque area. Macaque PEc indeed contains visual cells with a higher distribution of receptive fields in the lower VF, although the VF representation was not retinotopically organized (Gamberini et al. 2018, 2020). As highlighted by the conjunction analyses, our results suggest that the lower VF preference observed in hPEc overlaps with both hand and foot (but not eye) representations. This result supports the lack of a somatotopic organization of hPEc, again in line with macaque PEc (Gamberini et al. 2020) as well as with our previous report (Pitzalis et al. 2019) showing overlapping arm- and leg-related response in this area.

Macaque and human PEc share many other similar functional properties. Macaque area PEc contains bimodal visual/somatosensory neurons, neurons with large visual RFs, neurons with bilateral somatosensory RFs, and with somatosensory RFs located in both the forelimb and hindlimb (Breveglieri et al. 2006; Gamberini et al. 2018, 2020). It shows reach-related neural activity during visually guided reaching tasks in both 2D and 3D space (Battaglia-Mayer et al. 2001; Ferraina et al. 2001; Hadjidimitrakis et al. 2015; Piserchia et al. 2017) as well as a sensitivity to optic flow stimuli (Raffi et al. 2002, 2008). In addition, monkey PEc has visual neurons preferring optic flow with curve trajectories compatible with heading changes (Battaglia-Mayer et al. 2001; Raffi et al. 2002, 2010), and similar properties have been found in the newly defined region hPEc. We have previously observed that hPEc, like macaque PEc, exhibits somatosensory, visuomotor, and visual properties. It responds to both arm and leg movements, to both hand and foot pointing movements (Pitzalis et al. 2019), and to actual (but not imagined) grasping movements (Sulpizio et al. 2020), suggesting that it is involved in implementing the sensorimotor transformations needed to actually perform the action. Another striking similarity with the monkey counterpart is that the sensorimotor hPEc is involved in visual motion and optic flow processing. It indeed responds to flow field visual stimulation (Pitzalis et al. 2019) and, in a recent fMRI human study testing self- and object-motion in a naturalistic vision (Pitzalis et al. 2020), we have found that hPEc has a reliable preference for pure self-motion simulating continuous changes in heading direction. In other recent studies, we found that hPEc shows a visual preference for a curved path compared to a linear path (Di Marco et al. 2021). Notably, hPEc is able to integrate visual and somatomotor cues, with a preference for incongruent combination likely to signal a mismatch between these multisensory signals with the aim of promoting adjustments in lower limb movements during locomotion (Bellagamba et al. 2019). These last results suggest the presence of bimodal neurons in hPEc and reinforce the hypothesis suggested in Pitzalis et al. (2019) that area hPEc likely integrates visually derived self-motion signals with motor leg movement with the aim of guiding locomotion. Accordingly, both human and macaque PEc are functionally connected with the sensorimotor cortex representing lower limbs (Pitzalis et al. 2019; Bakola et al. 2010).

Overall, functional data on both species conclude in favor of area PEc as a cortical region specialized in controlling movements of both upper and lower limbs, as well as in their interaction with the visual environment. In agreement with this view, it has been shown that posterior parietal cortex (PPC) does not contain a strict effector-specify but rather an active representation common across effectors (Heed et al. 2011; Leone et al. 2014). More generally, previous studies suggested that PPC may participate in the control of visually guided voluntary movements and in their planning. For example, different regions of the monkey PPC (areas 5a, 5b, and 7) have received particular attention because of their role in multimodal integration and the fact that cells in these regions discharge during tasks requiring visuomotor transformation (Andersen and Buneo 2002; Andersen et al. 1997; Burnod et al. 1999; Jeannerod et al. 1995; Johnson et al. 1996; Mountcastle 1995; Mountcastle et al. 1975; Wise et al. 1996). In addition, in instructed delay tasks, in which information about the upcoming movement is provided before the signal to move, many cells in regions of the PPC related to reaching or grasping show strong anticipatory activation (Jeannerod et al. 1995; Kalaska 1996; Kalaska and Crammond 1995; Sakata et al. 1997; Snyder et al. 1997, 2000; Breveglieri et al. 2014; Santandrea et al. 2018). Here we extend this corpus of evidence by showing that a specific region within the human PPC, area hPEc, is further modulated by visuospatial features as the spatial location towards the upcoming hand and foot pointing was directed, similar to the monkey counterpart (Gamberini et al. 2018, 2020).

### Lower VF bias in the contralateral hemifield during foot pointing in areas hPE and S-I

Another result of this study is that the cortical territory located anteriorly to hPEc (corresponding to the two leg-related areas hPE and S-I) showed a significant interaction between vertical and horizontal dimensions of VF, as indicated by a preference for foot pointing directed to the lower relative to the upper VF only in the right (contralateral) hemifield and by a stronger response for foot pointing directed to contralateral relative to ipsilateral targets located in the lower VF.

The presence of a vertical asymmetry only in the contralateral hemifield is compatible with the somatotopic organization of these two regions, which predominantly represent the contralateral part of the body. In Pitzalis et al. (2019), we suggested that hPE likely corresponds to the medialmost part of a high-level parietal homunculus, found in the anterior SPL territory and called Parietal Body Area (PBA; Huang et al. 2012). The PBA contains a rough somatotopic representation of the entire body, with the lower limbs represented medially at the level of the postcentral sulcus (where hPE is located). The portion of S-I described here is anterior to hPEc and fits with the medial end of the human S-I, where the foot is somatotopically represented (Di Russo et al. 2006; Golaszewski et al. 2006; Huang et al. 2012; Akselrod et al. 2017). Our results, so far, provided indirect evidence in support of the somatotopic organization of hPE and S-I, since these two regions (unlike hPEc) showed a strict effector-selective response, both in the motor (responding to leg but not to arm movements) and visuomotor (responding to foot but not hand pointing) task (Pitzalis et al. 2019) as expected based on the somatotopic organization of PE (Taoka et al. 1998, 2000; Padberg et al. 2007; Seelke et al. 2012) and S-I (Di Russo et al. 2006; Golaszewski et al. 2006; Huang et al. 2012; Akselrod et al. 2017; Huang and Sereno 2018).

The evidence found here that brain activation elicited in hPE and S-I by limb movements is possibly modulated by the retinal position of the target is in line with recent human research which provided evidence for multisensory response properties of the somatosensory cortex (Kayser 2010). For instance, both hPE and S-I exhibited significant response to optic flow patterns simulating self-motion along a curved path (Di Marco et al. 2021). Similarly, also other fMRI studies found that the S-I activity is modulated not only by direct somatosensory input but also by visual stimulation (Dionne et al. 2010; Zhou and Fuster 1997; Kuehn et al. 2014, 2018). In particular, Kuehn et al. (2018) showed that fine-grained finger maps in human S-I, area 3b, are somatotopically activated not only during tactile mechanical stimulation, but also when seeing the same fingers being touched. The visually induced maps overlapped with the tactile maps providing evidence of the presence of somatotopically organized “foreign source maps” in early sensory cortices of the human brain. Here we extended these findings by showing that the activity in the somatomotor cortex is also involved in visuomotor control being strongly activated by lower limb movements directed towards the contralateral lower VF. Although caution is needed in the interpretation of the hPE and S-I results (see below), these data might reflect the role of this portion of cortex in providing highly topographically organized signals, likely useful to achieve an appropriate foot posture during locomotion.

### Lower VF preference for pointing movements in PMd

Beyond parietal regions (hPEc, hPE and S-I), we found a preference for actions directed towards the lower VF (compared to the upper VF) also in the frontal cortex, in a region located in the dorsal premotor cortex (PMd). Similarly to PEc, this area is modulated by both direction and depth information during reaching tasks, with the former encoded early during the target cue or movement planning period (Hadjidimitrakis et al. 2015; Fu et al. 1993, 1995; Messier and Kalaska 2000). Anatomical connections in macaque (Bakola et al. 2010) revealed that premotor area F2 (corresponding to the human PMd) forms the main motor connection of area PEc. In particular, PEc is more strongly connected with the sectors of motor and premotor cortex representing the lower limb than with those representing the upper limb (see Fig. 4C in Gamberini et al. 2020). A recent study of functional connectivity (Pitzalis et al. 2019) confirms this coupling also in humans. Moreover, it has been found that PMd (and the adjoining superior frontal sulcus or SFS) also contains primarily lower visual field representation adjoined with foot representation (Huang et al. 2012; Huang and Sereno 2018), similarly to the above-mentioned PBA. Overall, previous and present data suggest that hPEc and PMd might cooperate to coordinate planning and execution of goal-directed limb movements, likely in the lower-body space, e.g., when watching one’s steps.

### Specialization of the lower VF for visuomotor control

The current findings complement previous evidence of a functional specialization of the lower VF for the analysis and execution of visuomotor responses. Rossit et al. (2013) used slow event-related fMRI to examine such lower VF preference during reach-to-grasp hand movements. By manipulating the target position independently of the arm movement direction, they found that the superior parieto-occipital cortex and the precuneus were significantly more activated for object-oriented actions directed towards the lower relative to the upper VF. We found a similar specialization for visuomotor control in the lower VF within the precuneate cortex, particularly when participants performed delayed hand- and foot-pointing movements. Unlike Rossit et al. (2013), we did not find a lower VF preference in the superior parieto-occipital sulcus, where the human region responding to grasping/pointing movements (human V6A or hV6A) is located (Pitzalis et al. 2013; Tosoni et al. 2015; Sulpizio et al. 2020). We believe that this discrepancy is due to task differences, as subjects in Rossit et al. (2013) performed hand/fingers movements to actually interact with a real object while in the current study subjects were instructed to execute eye/hand/foot short-range pointing movements with no interaction with the target. Future studies are needed to understand whether areas hV6A and hPEc play independent or complementary roles in using their lower VF specialization for controlling different classes of hand/foot actions to interact with the surrounding objects.

As previously suggested by Rossit and colleagues (2013), the functional advantage of lower VF during visually guided actions might reflect the greater availability within the lower VF (compared to the upper VF) of visual information required for controlling limb movements (e.g., Graziano et al. 2004). The same functional specialization might be relevant to guide limb interaction with the environment and to correct foot posture, as needed during locomotion, especially if considering that the surface on which we locomote (the ground plane) typically falls in the lower VF. Notably, also the optic flow contains ecological specificities in the lower VF. Calow and Lappe (2007) found that visual motion properties of optic flow vary depending on visual field position such that some parts of the VF carry more information about ego-motion and others carry more information about depth. For example, velocities of natural optic flow fields in the lower VF are more tightly linked to the direction in which we move during walking (heading) than optic flow velocities in the upper VF. Thus, also evidence from the analysis of the local statistics of retinal optic flow supports the functional hypothesis of the lower visual field tightly linked to locomotion and this could be a window into the function of area hPEc.

Alternative or complementary explanations for the lower VF bias observed here during limb movements could be advanced. First, this result could be explained by differences in movement directions. Although the movement biomechanics did not remain constant during the experiment, participants used different effectors to point towards the memorized target location. The absence of a significant effector by VF interaction suggests that the lower VF preference is independent of the effector used (and thus of the specific movement performed) and allowed us to rule out the possibility that the observed activations are due to the specific movement biomechanics rather than on the spatial location towards the upcoming pointing movement was directed. We are aware that this last consideration applies only to hPEc and not to hPE and S-I, since in these two regions the lower VF preference was found only in the foot pointing task. However, note that whole-brain results confirmed the absence of an effector by VF interaction in all the investigated regions. Additionally, it is also unlikely that our results are explained by low-level differences in movement parameters since they were minimized by asking participants to perform very short-range pointing movements. Second, an alternative explanation is that an attentional modulation might drive the observed results. Participants were instructed to perform a delayed pointing task which required the allocation of attentional resources to attend to a specific spatial location. It has been suggested that the lower VF has better attentional resolution than the upper VF (e.g., He et al. 1996; Rubin et al. 1996; Ellison and Walsh 2000; Talgar and Carrasco 2002; for review see Danckert and Goodale 2003). Specifically, regions belonging to the dorsal frontoparietal attentional network (e.g., Corbetta et al. 2008), such as the anterior and posterior segments of the intraparietal sulcus (IPs), the frontal eye fields and a region at the junction between the posterior IPs and the transverse occipital sulcus, have been described as showing a lower VF preference during stationary spatial orienting and an upper VF preference during visual search (Kraft et al. 2011). However, contrary to these findings, we did not observe any significant VF preference in these regions, thus suggesting that the lower VF preference here observed in the dorsomedial parietal cortex during limb actions is unlikely to be entirely driven by attention. Finally, a neural modulation as a function of the shift of attention has been observed in the medial posterior area V6A (Galletti et al. 2010; Ciavarro et al. 2013; Caspari et al. 2015), but not in the cortical territory anterior to it.

### Reference frames for eye/hand/foot pointing movements

During the last 20 years several neurophysiological and neuroimaging studies have compared eye and hand/foot movements to demonstrate that distinct neural circuits are preferentially active for movements involving different body parts (Graziano and Gross 1998; Andersen and Cui 2009; Caminiti et al. 2010; Galati et al. 2011; Tosoni et al. 2015; Heed et al. 2011). Although this is a very common and standard practice and we used it ourselves in this study, it should be noted that there are computational differences in the frame of reference between eye and hand/foot pointing movements execution. Indeed, while the motor vector for saccade is directly provided by the retinotopic coordinates of the target, the motor vector for hand and foot pointing movements requires computing the spatial relationship between limb and target location, a computation that rely on an estimate of the body’s current posture and that requires an alignment of the reference frames involved in visual processing and limb movement execution. This is why, whereas saccade is supposed to occur in eye-centered coordinates, limb movement is suggested to use both eye- and body-related reference frames (Pesaran et al. 2006, 2010; Marzocchi et al. 2008; Chang et al. 2009; Ferraina et al. 2009; McGuire and Sabes 2009; Bernier and Grafton 2010; Chang and Snyder 2010; Piserchia et al. 2017).

A way to disentangle the contribution of eye- and body-related reference frames would be to dissociate the visual target from the location pointed by the effector. Unfortunately, unlike previous studies (e.g., Rossit et al. 2013), we did not manipulate the position of the fixation point, thus we cannot dissociate the egocentric spatial location of the target by its retinal location. Further investigations are needed to disambiguate the actual contribution of eye- and body-related reference frames and to establish whether the observed lower VF preference in the dorsomedial parietal cortex is related to a bias in the sensory representation of the target and/or to a bias in its motor repertoire.

However, while acknowledging the possible differences between eye and hand/foot pointing movements execution, it should be noted that our hypotheses (and analyses) mainly focused on hand and foot pointing conditions, which rely on a similar reference frame.

### Conclusions

It has been suggested that the lower visual field is important for guiding locomotion in the presence of ground obstacles (Marigold 2008; Marigold and Patla 2008). In accordance with this observation, we tentatively consider locomotion as one of the foremost functions of hPEc because its preference for actions directed towards the lower visual field should be particularly useful for the online control of the coordination of limb movements, especially over uneven terrains. Beyond hPEc, also the cortical territory hosting foot-selective representations (corresponding to the areas hPE and S-I) exhibits a preference for foot movements directed towards the lower visual field, although only in the contralateral visual hemifield. This might suggest a role of this portion of cortex in providing topographically organized somatotopic signals, likely useful to achieve an appropriate foot posture during locomotion.

## Supplementary Information

Below is the link to the electronic supplementary material.

Supplementary file1 (DOCX 50 KB)

Supplementary file2 (TIFF 18986 KB)
