# Supplementary material for: Lower visual field preference for the visuomotor control of limb movements in the human dorsomedial parietal cortex
Source: Brain Struct Funct. 2021 Mar 18;226(9):2989–3005. doi: 10.1007/s00429-021-02254-3 (PMC8541995; doi:10.1007/s00429-021-02254-3)
Supplement: Supplementary file 1 — Supplementary file1 (DOCX 50 KB) [file 429_2021_2254_MOESM1_ESM.docx]

*Supplementary Figure 1. Group maps showing significant interaction between effector and dimensions of VF.* Whole-brain activation maps showing effector by vertical dimension interaction (**A**) and effector by vertical by horizontal dimension interaction (**B**).

Supplementary Table 1. Information about target locations: eight possible target locations are described as a function of their eccentricity, the angle of rotation, XY coordinates (in deg of visual angle) and the portion of VF (lower or in the upper) they appeared.

| *Target position* | *Eccentricity* | *Angle* | *Target Position X* | *Target Position Y* | *VF* |
| --- | --- | --- | --- | --- | --- |
| 1 | 4 | 30 | 3.46 | 2.00 | upper right |
| 2 | 4 | 60 | 2.00 | 3.46 | upper right |
| 3 | 4 | 120 | -2.00 | 3.46 | upper left |
| 4 | 4 | 150 | -3.46 | 2.00 | upper left |
| 5 | 4 | 210 | -3.46 | -2.00 | lower left |
| 6 | 4 | 240 | -2.00 | -3.46 | lower left |
| 7 | 4 | 300 | 2.00 | -3.46 | lower right |
| 8 | 4 | 330 | 3.46 | -2.00 | lower right |
