# Supplementary figures and images for: Lower visual field preference for the visuomotor control of limb movements in the human dorsomedial parietal cortex
Source: Brain Struct Funct. 2021 Mar 18;226(9):2989–3005. doi: 10.1007/s00429-021-02254-3 (PMC8541995; doi:10.1007/s00429-021-02254-3)

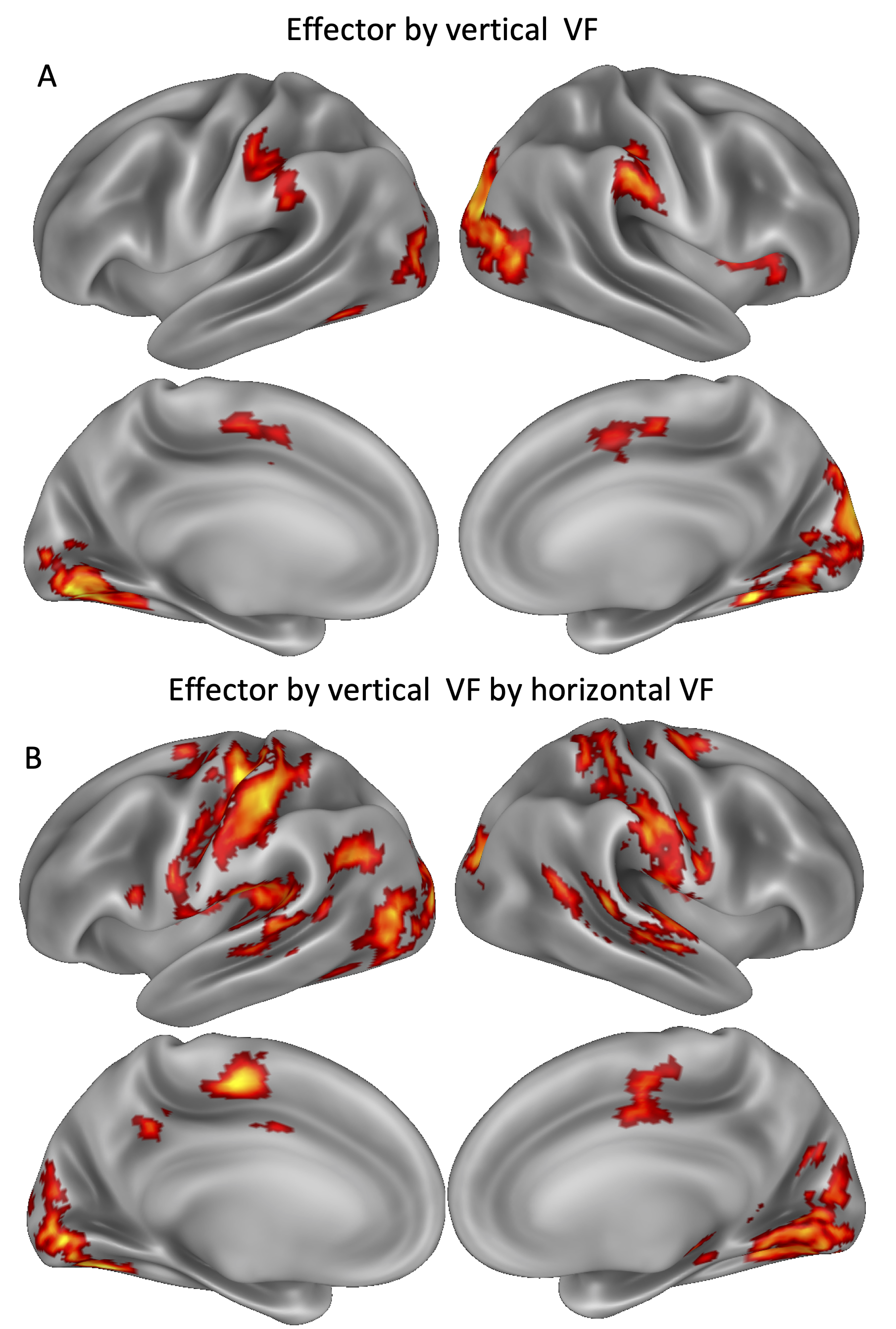

Supplement: Supplementary file 2 — Supplementary file2 (TIFF 18986 KB) [file 429_2021_2254_MOESM2_ESM.tiff]
